# Decreased serum iron concentration and total iron binding capacity are associated with serious Crohn’s disease

**DOI:** 10.1038/s41598-022-07948-0

**Published:** 2022-03-10

**Authors:** Jingling Su, Yandan Ren, Lupeng Liu, Yiqun Hu, Huaxiu Shi, Jianlin Ren, Chenxi Xie

**Affiliations:** Department of Gastroenterology, Zhongshan Hospital Xiamen University, Xiamen, 361000 Fujian Province China

**Keywords:** Gastroenterology, Gastrointestinal diseases

## Abstract

This study aimed to investigate whether serum indicators related to iron stores in the body are associated with clinical and endoscopic disease severity. Eighty-four patients with Crohn’s disease (CD) and twenty-four healthy volunteers were included. The indicators related to iron stores were detected within one week after endoscopic and CT enterography examinations. Patients were divided into three groups according to the CDAI(Crohn's disease activity index)scores. Serum iron levels were decreased in all groups (*p* < 0.05), and the values of remission group were higher than those of moderate group (*p* < 0.001). The total iron binding capacity(TIBC)values of the moderate group were lower than those of the controls and the other groups (*p* < 0.05). None of the indicators differed significantly among the patients classified by SES-CD (*p* > 0.05). Underweight, decreased serum iron and TIBC were independent risk factors for moderate clinical disease. Combined detection of decreased serum iron and TIBC was helpful in differentiating severe patients. The sensitivity and specificity were 32.7% and 100%, respectively (AUC = 0.812, *p* < 0.01). Decreases in serum iron and TIBC were associated with the clinical activity of CD. Combined detection of the two indicators was conducive to screening serious disease.

## Introduction

Patients with Crohn’s disease (CD) develop various extraintestinal complications. Iron deficiency can be encountered in 13%-90% of patients according to previous reports^1,2^. Iron deficiency plays a key role in affecting the quality of life of CD patients, resulting in symptoms, such as anemia, fatigue, sleep disorders, attention deficits and reduced functional capacity^3,4^. Factors leading to iron deficiency include bleeding, reduced iron absorption and immune regulation.

Cytokines, such as IL-6 and TNF-a, can alter serum iron parameters by regulating the expression of hepcidin^5–7^. The production of active hepcidin by the liver is increased upon stimulation by cytokines from the inflamed bowel. Hepcidin can bind to the iron exporterferroportin1 on the basolateral membrane of enterocytes, leading to protein internalization and degradation^8,9^, this limits the efflux of iron to the plasma. Overload of iron content in enterocytes reduces the expression of duodenal cytochrome B (DcytB) and divalent metal transporter 1 (DMTI) on the brush border membrane, inhibiting dietary iron absorption^9^. Another important source of hepcidin is dendritic cells (DCs) in the gut^10^. In the absence of DC-derived hepcidin, iron can be released from macrophages and neutrophils to the lumen through ferroportin^10^. Basseri RJ et al. found that serum hepcidin was positively correlated with IL-6 and negatively correlated with hemoglobin in anemia of chronic disease (ACD)^11^.This suggested that iron stored in these inflammatory cells is an important source of hematopoiesis in CD patients with ACD.

Although hepcidin is a key mediator of anemia in active CD patients, it is not tested routinely, partially because it may be difficult to distinguish active or inactive forms by commonly used immunoassays^12^. Semrin G et al. measured iron status in young CD patients. They showed that the level of serum iron and the total iron binding capacity were decreased in active CD patients due to impaired oral iron absorption^8^. Therefore, we hypothesized that the indicators related to serum iron parameters may be candidates for evaluating disease severity.

Thus, the aims of the current study are (1) to compare the differences in serum indicators related to iron stores in the body between CD patients and controls and (2) to investigate whether these indicators are associated with disease severity.

## Results

### Demographic characteristics of the patients

A total of one hundred twenty-nine CD patients who were first admitted to our hospital were screened. Seventeen with a history of intestinal surgery and twenty-eight unable to tolerate endoscopy or CTE examination were excluded from this study. However, four who underwent laparoscopic appendectomy were included. Ultimately, eighty-four patients and twenty-four healthy volunteers were enrolled in the analysis (Table 1).

**Table 1:** Demographic data of the patients and controls.

	CD patients (n = 84)	Controls (n = 24)	*p* value
Age (years)	28 (22,33.75)	47 (39.50,51.75)	0.00
Male (%)	64.29%	45.83%	0.103
BMI (kg/m^2^)	18.96 (17.14,21.44)	22.89 (20.90,26.65)	0.00

BMI: body mass index.

For age and BMI: Wilcoxon rank-sum test.

For gender distribution: Chi square test.

## Comparison of the indicators related to serum iron parameters among patients classified by CDAI scores and controls

The patients were divided into three groups according to the CDAI scores. None with severe activity could be found in our study (Table 2).

**Table 2:** Comparison of the serum indicators among patients classified by CDAI scores and controls.

	Remission (n = 15)	Mild (n = 16)	Moderate (n = 53)	Control (n = 24)	*p* value
Iron (µmol/L)	10.50^a,b^ (8.50,14.30)	7.65^a^ (5.55,10.78)	5.60^a^ (3.80,9.60)	16.15 (14.15,22.25)	0.00
TIBC (µmol/L)	53.29 ± 11.58^b^	52.23 ± 13.01^b^	43.67 ± 9.34^a^	53.58 ± 9.81	0.00
Ferritin (ug/L)	124.80 (56.80,200)	66.60 (39.85,187.20)	88.10 (47.95,212.75)	207.65 (62, 323.68)	0.182
Transferrin (g/L)	2.32^b^ (1.91,2.72)	2.12 (1.78,2.67)	1.87^a^ (1.62,2.12)	2.15 (2.03,2.50)	0.00
Transferrin saturation (%)	27 (15,33)	16^a^ (11,22.25)	14^a^ (9, 21.75)	32 (25.5,44)	0.00

TIBC: total iron binding capacity.

For TIBC: One-way ANOVA.

For the other parameters: Kruskal–wallis H test.

*p* < 0.05 means that the distribution of values in each group is not equal.

The letter a means that the difference is significant when compared to the controls separately.

The letter b means that the difference is significant when compared to the moderate group separately.

The serum iron concentration was decreased in all the groups (*p* < 0.05). The values of the moderate group were lower than those of the remission group (*p* < 0.001), but the difference was not significant between the remission and mild groups (*p* > 0.05).

The level of TIBC was decreased significantly in the moderate group compared with controls and the other groups (*p* < 0.05), but the values were similar between controls and the other groups (*p* > 0.05).

The transferrin values of the moderate group were lower than those of the remission group and the controls (*p* < 0.05). The difference was not significant between the other groups and the controls (*p* > 0.05).

Transferrin saturation was lower in the mild and moderate groups than in the controls (*p* < 0.05). However, the values did not differ significantly among the three groups (*p* > 0.05).

There was no significant difference in ferritin values between CD patients and controls (*p* > 0.05).

### Comparison of the indicators related to serum iron parameters among patients classified by SES-CD and controls

The patients were divided into four groups according to the SES-CD. The results of these comparisons were shown in Table 3.

**Table 3:** Comparison of the serum indicators among patients classified by SES-CD and controls.

	Remission (n = 13)	Mild (n = 17)	Moderate (n = 34)	Severe (n = 20)	Control (n = 24)	*p* value
Iron (µmol/L)	8.8^**a**^ (6.40, 11.75)	7.60^**a**^ (5.65,10.70)	8.25^**a**^ (4.65,11.85)	4.70^**a**^ (3.75,8.20)	16.15 (14.15,22.25)	0.000
TIBC (µmol/L)	48.88 ± 12.98	48.73 ± 11.96	46.66 ± 10.76	45.22 ± 11.06	53.58 ± 9.81	0.119
Ferritin (ug/L)	65.90 (31.95,154.00)	124.80 (39.30,204.00)	126.35 (60.20,211.90)	68.90 (40.63,186.30)	207.65 (62,323.68)	0.147
Transferrin (g/L)	1.95 (1.67,2.44)	2.12 (1.65,2.58)	1.98 (1.68,2.31)	1.86 (1.65,2.52)	2.15 (2.03,2.50)	0.049
Transferrin saturation (%)	21^**a**^ (10, 29.5)	17.50^**a**^ (11.75,31.50)	17.50^**a**^ (9.50,26)	11.50^**a**^ (9, 16.75)	32 (27,44)	0.000

TIBC: total iron binding capacity.

For TIBC: One-way ANOVA.

For the other parameters: Kruskal–wallis H test.

*p* < 0.05 means that the distribution of values in each group is not equal.

The letter a means that the difference is significant when compared to the controls separately.

The values of serum iron and transferrin saturation were lower in all the groups than in the controls (*p* < 0.05). However, the values did not differ significantly among the four groups (*p* > 0.05).

Although there was a decreasing trend of transferrin values in all the groups (*p* = 0.049), the differences were not significant when compared with controls separately (*p* > 0.05).

There were no significant differences in ferritin and TIBC values between CD patients and controls (*p* > 0.05).

The results suggested that there was no correlation between these indicators and lesion severity under endoscopy.

### Association of the indicators related to serum iron parameters with clinical disease severity

We found that serum iron, TIBC and transferrin were significantly decreased in patients with moderate clinical disease, but the differences were not significant between the mild and remission groups. These results suggested that these indicators may be candidates for evaluating disease severity.

The cutoff value of serum iron used to distinguish moderate from mild/remission groups was 5.25 µmol/L (AUC = 0.729, *p* = 0.001), the sensitivity was 48.1%, and the specificity was 93.5%.

The cutoff value of TIBC used to distinguish moderate from mild/remission groups was 47.25 µmol/L (AUC = 0.723, *p* = 0.001), the sensitivity was 73.1%, and the specificity was 67.7%.

The cutoff value of transferrin used to distinguish moderate from mild/remission groups was 2.07 g/L (AUC = 0.708, *p* = 0.003), the sensitivity was 75%, and the specificity was 64.5%.

The three cutoff values above were used to divide all the CD patients into two groups separately. Patients were classified by age, disease location or behavior according to the Montreal classification^13^. A body mass index (BMI) < 18.5 kg/m^2^ was used as the demarcation of underweight^14^. Hemoglobin values of < 130 g/L for males and < 120 g/L for females were used as the demarcations of anemia^15^. The comparison results of these metrics between two groups were shown in Table 4.

**Table 4:** Comparison of metrics between CD patients with different disease severities.

	Mild/Remission (n = 31)	Moderate (n = 53)	*p* value
**Age (years)**			0.037
≤ 16 (n)	7	4	
17–40 (n)	22	41	
> 40 (n)	2	8	
Male (n)	21	33	0.613
BMI (< 18.5 kg/m^2^)	5	31	0.00
**Location (n)**			
Ileal	4	3	0.680
Colonic	2	4	
Ileocolonic	18	31	
UGIT involved	7	15	
**Behavior (n)**			0.116
Non‐stricturing, non‐penetrating	12	14	
Structuring	12	22	
Penetrating	5	4	
Stricturing and penetrating	2	13	
With anemia (n)	16	46	0.00
Iron(< 5.25 µmol/L) (n)	2	26	0.00
TIBC (< 47.25 µmol/L) (n)	10	39	0.00
Transferrin (< 2.07 g/L) (n)	11	39	0.001
Transferrin saturation(%)	18 (14,28)	14 (9,21.75)	0.042
Ferritin (µg/L)	114.80 (49,196)	88.10 (47.95,212.75)	0.774

BMI: body mass index.

UGIT: upper gastrointestinal tract.

TIBC: total iron binding capacity.

For age, BMI, iron, TIBC and transferrin: rank-sum test.

For gender distribution, location, behavior and anemia: Chi square test.

For transferrin saturation and ferritin: wilcoxon rank-sum test.

Only the parameters with statistical differences between groups were included in the regression analysis.

Logistic regression analysis was applied to identify the potential risk factors for serious Crohn’s disease. The results showed that underweight, decreased serum iron and TIBC were independent risk factors for serious disease (for underweight, OR = 7.344, 95% CI 1.868–28.875, for decreased serum iron, OR = 21.315, 95% CI 3.525–128.88, for decreased TIBC OR = 8.259, 95% CI 2.267–30.086).

We found that serum iron and TIBC correlated negatively with CDAI scores (r = − 0.513, r = − 0.409, both *p* < 0.05).

In addition, the combined use of decreased serum iron and TIBC was effective in distinguishing serious disease, with a sensitivity of 32.7% and a specificity of 100% (AUC = 0.812, *p* < 0.01).

## Discussion

Crohn’s disease is a recurrent disorder of the gastrointestinal tract, often accompanied by complicated extraintestinal manifestations. Iron deficiency (ID) and iron deficiency anemia (IDA) are frequently encountered in up to 90% of IBD patients due to chronic active illness^2,16^. Oral iron would be ineffective in patients with higher CRP, and is harmful to mucosal healing^10,17,18^. Oral iron therapy was associated with decreased abundance of Faecalibacterium prausnitzii*,* Ruminococcus bromii*,* Dorea *sp.* and Collinsella aerofaciens^19^. Reduction in intestinal probiotic community leads to the invasion and colonization of pathogenic bacteria. Intestinal iron accumulation can aggravate inflammation by suppressing Bifidobacterium species^18^, and is associated with serum iron deficiency. In this study, we evaluated the association between serum indicators related to iron stores and disease severity and found that the detection of decreased serum iron and total iron binding capacity was effective in screening serious disease.

In this study, serum iron concentration and TIBC were decreased significantly in moderate clinical disease. This is consistent with the progression in Crohn’s disease. CD can affect any region within the gastrointestinal tract. Chronic bleeding caused by segmental ulceration or mucosal inflammation predisposes to lose massive amounts of iron^3,4^. The involvement of the duodenum-jejunum and reduced daily food intake due to fear of gastrointestinal symptoms can aggravate iron malnutrition^4^. Furthermore, the complex interplay of cytokines produced by the inflamed intestine and the surrounding mesentery and hepcidin can also contribute to iron deficiency^1,20^. Two major relevant sources of hepcidin are hepatocytes and dendritic cells in the intestinal tract^10,17^. Functionally active hepcidin from the liver is upregulated by cytokines and binds to ferroportin on enterocytes via the blood circulation, inducing degradation of this iron transporter^9^. In the absence of DC-derived hepcidin during inflammation, ferroportin in macrophages and neutrophils is retained, and iron is released extracellularly^10^. These changes limit iron absorption and transport to the plasma. Systemic inflammation had a significant impact on serum iron homeostasis. This was in line with our results showing that serum iron and TIBC correlated negatively with CDAI scores, and the specificity of decreased serum iron was very high in distinguishing moderate CD. Combined detection of these two indicators would be useful in screening serious CD.

Ferritin is a measure of stored iron content and is decreased in the condition of iron deficiency^21^. Previous studies reported that ferritin was positively correlated with hepcidin and negatively correlated with the efficacy of oral iron treatment^5,22^. These results suggested that ferritin may be increased due to active intestinal inflammation^23^. For CD, severe inflammation is often accompanied by iron deficiency, and false normal ferritin may be found^4^. This may explain why the difference in ferritin was not significant between patients and controls in our study. Both serum ferritin and transferrin are active-phase reactants^9^. Transferrin is responsible for transferring iron from the sites of absorption to all tissues. The level is high in patients with iron deficiency, but it will decrease during inflammation^9^. Therefore, false normal values were commonly found in the condition of anemia of inflammation. In our study, although transferrin and transferrin saturation seemed lower in moderate CD, neither of them was useful in screening serious disease.

We found that disease localization and behavior were not associated with severity. This was consistent with the study of Aksan A et al^5^. It seemed that serum iron concentration and transferrin saturation tended to decrease in patients with higher SES-CD, but the differences were not significant. This result was reasonable because SES-CD is based on colonoscopy findings and cannot reflect lesions beyond the stricture or in the upper gastrointestinal tract^24^. Although ulcers are an important indicator of SES-CD, iron deficiency is not only associated with ulceration or bleeding^4,15,25,26^.

Dietary Fe intake is reduced in CD patients as a result of avoidance of certain fiber-rich and Fe-fortified cereals due to the fear of exacerbating gastrointestinal symptoms^4^.Active disease can decrease absorption and increase energy expenditure^27,28^. Combined, these factors contribute to the weight loss observed. Undernutrition has a negative impact on the disease process and increases the rate of postoperative complications and mortality^28–31^. BMI<18.5kg/m^2^ is one of the criteria for undernutrition^27^. It was not surprising to find that this metric was an indicator for serious Crohn’s disease.

There are some limitations in this study. First, age was not comparable between CD group and controls. This may have a negative impact on the reliability of our conclusion. Therefore, logistic regression analysis was used to exclude the influence of confounding factors, and we found that decreased serum iron and total iron binding capacity were correlated with clinical disease severity. Second, none with severe clinical activity were included in this study. This may be due to the improvement of patients’ health consciousness; they will seek medical advice in time as gastrointestinal symptoms appear. We will include more patients in a future study and try to obtain data from group with severe disease. Third, this was a single-center prospective study, and the sizes of groups were small after classification based on the CDAI scores or SES-CD. We described the observed results but lacked data on patients after immunotherapy and iron supplementation. Further study on the correlation between the changes in these indicators and that of CDAI scores after treatment would be useful in supporting our conclusion.

## Conclusion

In summary, our findings suggested that decreased serum iron and total iron binding capacity were associated with the severity of Crohn’s disease. Decreased serum iron was less sensitive than TIBC in distinguishing moderate CD from the mild/remission group but showed higher specificity. The combined use of these two indicators for screening serious disease would be helpful in developing targeted treatment programs.

## Materials and Methods

### Subjects

Consecutive patients diagnosed with Crohn’s disease (CD) in our hospital from February 2018 to November 2020 were enrolled in the prospective study. Patients were excluded if they had the following conditions: unable to undergo colonoscopy and CT enterography (CTE) examinations due to severe intestinal obstruction or perforation; gastrointestinal tumors; previous abdominal surgery (except laparoscopic appendectomy);other systemic diseases that can lead to anemia; received oral/intravenous iron treatment or blood transfusion within 3 months prior to the study; women in the pregnancy or breast-feeding stage; and severe renal, cardiac or pulmonary disease. Ultimately, eighty-four were included. Colonoscopy and CTE were performed for all within 1 week before blood samples were taken. Another 24 volunteers with normal mucosa via colonoscopy and without gastrointestinal symptoms, systemic disorders or major abdominal surgery were simultaneously recruited as healthy controls.

The study protocol and the recruitment of the patients and controls were approved by the Ethics Committee of Zhongshan Hospital Xiamen University (Ethical approval No: xmzsyyky 2021–166). Written informed consent was obtained from all individuals before starting any study procedure. We confirmed that all methods were performed in accordance with the relevant guidelines and regulations.

### Standards of grading for disease severity

Lesion location and disease behavior were recorded according to the Montreal classification. The Crohn’s disease activity index (CDAI) was used to assess clinical disease severity. In this study, CDAI < 150 suggested remission, 150–220 with mild activity, 221–450 with moderate activity, and > 450 with severe activity^32^.

The colonoscopy procedures were performed in our hospital by the same physician (Xie Chenxi). A complete colonoscopy should include the whole colon and enter 20 cm into the end of ileum. The withdrawal time should be more than 6 min. Biopsies should not be taken from the bottom of ulcer. The findings were recorded according to the simple endoscopic score of Crohn’s disease (SES-CD). Four endoscopic variables were scored from 0 to 3: size of ulcers (0 = none; ulcer diameter 0.1–0.5 cm = 1; 0.5–2 cm = 2; > 2 cm = 3); ulcerated surface(0 = none; < 10% = 1; 10%–30% = 2; > 30% = 3); surface affected by any lesions (0 = none; < 50% = 1; 50–75% = 2; > 75% = 3); and presence of narrowing (0 = none; single, can be passed = 1; multiple, can be passed = 2; cannot be passed = 3). SES-CD is the sum of these scores in the five segments (terminal ileum, right colon, transverse colon, left colon and rectum). In this study, SES-CD scores between 0 and 2 suggested remission, 3–6 mild activity, 7–15 moderate activity and ≥ 16 severe activity^33^.

### Assessment of indicators related to serum iron homeostasis

Five indicators, including serum iron (SI), total iron binding capacity (TIBC), ferritin, transferring (Tf), and transferrin saturation (TSAT%), were assessed in the study. These indicators are commonly used in clinical practice and are easy to detect. 2 ml blood samples were taken after fasting for at least 8 h. The serum and blood cells shall be separated within 2 h after collection, and the detection shall be completed within 8 h. If the process could not be completed in time, the serum samples should be stored at 2–8 °C. Serum iron and TIBC were detected by Beckmann AU5800 biochemical analyzer. Ferritin was detected by Roche Cobas e602 Automatic immune analyzer. Transferrin and TSAT% were detected by BN II specific protein analyzer. All assays were performed according to the instructions of relevant instruments by an investigator blinded to the case–control status.

### Statistical analysis

Data are expressed as either the mean ± SD or the median (interquartile range). One-way ANOVA was used to compare differences if the values for a metric followed normal distribution; otherwise, the rank-sum test was used. The Spearman rank correlation coefficient was used to analyze the correlation between CDAI scores and the levels of serum iron or total iron binding capacity. Logistic regression analysis was applied to investigate the association of serum indicators with clinical disease severity. A *p* value less than 0.05 was considered statistically significant. The statistical analysis was accomplished using SPSS20.0 (SPSS Inc., Chicago, IL, USA).

### Ethics approval and consent to participate

The study protocol and the recruitment of the patients and controls were approved by the Ethics Committee of Zhongshan Hospital Xiamen University. Approval No: xmzsyyky 2021–166. Written informed consent was obtained from all individuals before starting any study procedure.

## Data Availability

The datasets used and/or analyzed during the current study are available from the corresponding author on reasonable request.

## Abbreviations

CD: Crohn’s disease
CDAI: Crohn’s disease activity index
SES-CD: Simple endoscopic score of Crohn’s disease
CTE: CT enterography
DcytB: Duodenal cytochrome B
DMT1: Divalent metal transporter 1
DC: Dendritic cell
SI: Serum iron
TIBC: Total iron binding capacity
Tf: Transferrin
TSAT: Transferrin saturation
BMI: Body mass index
